# Human Omentin-1 Administration Ameliorates Hypertensive Complications without Affecting Hypertension in Spontaneously Hypertensive Rats

**DOI:** 10.3390/ijms24043835

**Published:** 2023-02-14

**Authors:** Yuta Okamura, Ryo Niijima, Satoshi Kameshima, Tomoko Kodama, Kosuke Otani, Muneyoshi Okada, Hideyuki Yamawaki

**Affiliations:** 1 Laboratory of Veterinary Pharmacology, School of Veterinary Medicine, Kitasato University, Higashi 23 Bancho 35-1, Towada 034-8628, Aomori, Japan; 2 School of Veterinary Medicine, Kitasato University Veterinary Teaching Hospital, Higashi 23 Bancho 35-1, Towada 034-8628, Aomori, Japan; 3 Laboratory of Small Animal Internal Medicine, School of Veterinary Medicine, Kitasato University, Higashi 23 Bancho 35-1, Towada 034-8628, Aomori, Japan

**Keywords:** hypertension, adipocytokine, systolic blood pressure, arterial contractility, heart failure, renal failure

## Abstract

Hypertension is one of the major risk factors for cardiovascular diseases and is caused by various abnormalities including the contractility of blood vessels. Spontaneously hypertensive rats (SHR), whose systemic blood pressure increases with aging, are a frequently used animal model for investigating essential hypertension and related complications in humans due to the damage of several organs. Human omentin-1 is an adipocytokine consisting of 313 amino acids. Serum omentin-1 levels decreased in hypertensive patients compared with normotensive controls. Furthermore, omentin-1 knockout mice showed elevated blood pressure and impaired endothelial vasodilation. Taken together, we hypothesized that adipocytokine, human omentin-1 may improve the hypertension and its complications including heart and renal failure in the aged SHR (65–68-weeks-old). SHR were subcutaneously administered with human omentin-1 (18 μg/kg/day, 2 weeks). Human omentin-1 had no effect on body weight, heart rate, and systolic blood pressure in SHR. The measurement of isometric contraction revealed that human omentin-1 had no influence on the enhanced vasocontractile or impaired vasodilator responses in the isolated thoracic aorta from SHR. On the other hand, human omentin-1 tended to improve left ventricular diastolic failure and renal failure in SHR. In summary, human omentin-1 tended to improve hypertensive complications (heart and renal failure), while it had no influence on the severe hypertension in the aged SHR. The further study of human omentin-1 may lead to the development of therapeutic agents for hypertensive complications.

## 1. Introduction

Hypertension is one of the major risk factors for cardiovascular diseases including coronary artery disease, left ventricular hypertrophy, valvular heart diseases, cardiac arrhythmias, cerebral stroke, and renal failure [1]. Hypertension is mainly caused by dysfunctional vascular reactivity and various changes in the structure and function of arteries such as vascular inflammation and remodeling via the proliferation and migration of smooth muscle cells [2]. Spontaneously hypertensive rats (SHR) whose systemic blood pressure increases with aging are frequently used animal models for investigating essential hypertension in humans [3]. Moreover, it was reported that the survival rate in SHR decreases after 54 weeks because of the severe damage found in several organs [4].

Adipocytes can secrete a variety of cytokines, termed adipocytokine. Adipocytokine is a biologically active molecule secreted from the adipose tissues [5]. Obesity with an accumulation of visceral fat is one of the major risk factors for cardiovascular diseases including hypertension. Adipocytes enlarged by obesity may increase or decrease the production and secretion of adipocytokines. Adipocytokine is thought to regulate obesity-related hypertension by directly acting on vascular function [6].

Human omentin-1 is an adipocytokine consisting of 313 amino acids [7]. It was reported that serum human omentin-1 levels decreased in hypertensive patients compared with normotensive controls [8]. Furthermore, omentin-1 knockout mice showed elevated blood pressure and impaired endothelial vasodilation [9]. We previously demonstrated that human omentin-1 caused vasodilation in rat isolated blood vessels via stimulating endothelial nitric oxide (NO) production [10]. In addition, we demonstrated that intravenously injected human omentin-1 acutely inhibited agonist-induced increase in the blood pressure in rats [11]. Moreover, we demonstrated that human omentin-1 treatment inhibited monocrotaline-induced pulmonary arterial hypertension in rats via inhibiting vascular structural remodeling and abnormal contractile reactivity [12]. It was also reported that human omentin-1 had a protective role against atherosclerotic plaque vulnerability [13] and myocardial ischemic injury [14].

Taken together, we hypothesized that human omentin-1 may improve hypertension and its complications including heart and renal failure in a late stage. To test the hypothesis, we investigated whether human omentin-1 improves (1) elevated blood pressure through inhibiting dysfunctional vascular reactivity and (2) complications including heart and renal dysfunction in aged SHR.

## 2. Results

### 2.1. Human Omentin-1 Had No Effect on Body Weight (BW), Heart Rate (HR), and Systolic Blood Pressure (SBP) in SHR

**Figure 1:**
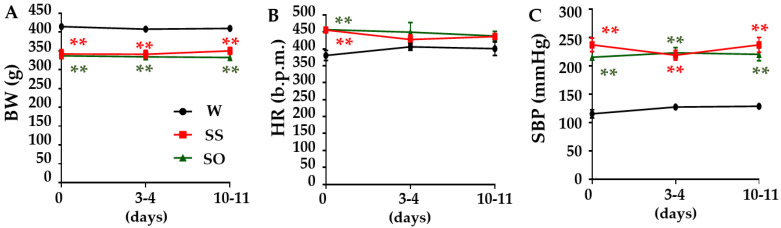
Human omentin-1 had no effect on body weight (BW), heart rate (HR), or systolic blood pressure (SBP) in spontaneously hypertensive rats (SHR). Male SHR (65–68-week-old) were subcutaneously injected with saline (0.14 mL/day, n = 4; SS) and human omentin-1 (18 μg/kg/day, n = 4; SO) by using an implanted osmotic pump for 14 days. Male Wistar Kyoto rats (WKY) were sham operated (n = 4; W). BW (**A**) was measured once a week. HR (**B**) and SBP (**C**) were measured by a tail-cuff method (before the injection (0) and 3–4 and 10–11 days after the injection). The results were expressed as means ± standard error of the mean (S.E.M.). b.p.m.; beats per minutes. ** *p* < 0.01 vs. W (two-way ANOVA followed by Holm–Sidak post-hoc test).

We first examined the effects of human omentin-1 on BW, HR, and SBP in SHR. The BW of SHR injected with saline (0.14 mL/day; SS) was significantly lower than sham operated Wistar Kyoto rats (WKY; W) (Figure 1A, *p* < 0.01, n = 4). Human omentin-1 (18 μg/kg/day; SO) did not affect the BW of SHR (Figure 1A, n = 4). While the HR of SHR (n = 8) at day 0 (before injection) was significantly higher than WKY (n = 4) (Figure 1B, *p* < 0.01), HR did not differ between groups after injection (at day 3–4 and day 10–11, Figure 1B, n = 4). The SBP of SS was significantly higher than W (Figure 1C, *p* < 0.01, n = 4). Human omentin-1 (18 μg/kg/day, n = 4) did not affect the SBP of SHR (Figure 1C).

### 2.2. Human Omentin-1 Had No Effect on Agonist-Induced Contraction and Relaxation in Isolated Thoracic Aorta

**Figure 2:**
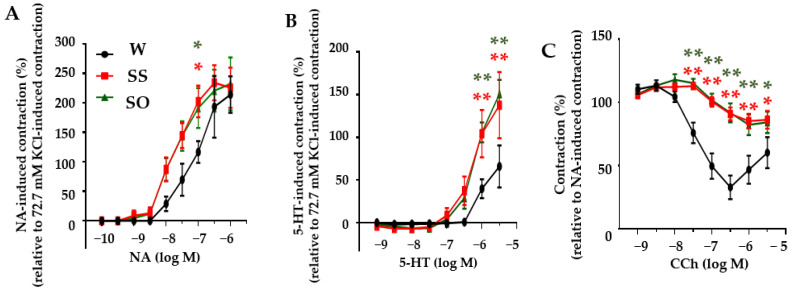
Human omentin-1 had no effect on agonist-induced contraction and relaxation in isolated thoracic aorta. The thoracic aorta was harvested from male SHR (65–68-week-old) subcutaneously injected with saline (0.14 mL/day; SS) and human omentin-1 (18 μg/kg/day; SO) by using an implanted osmotic pump for 14 days or sham operated WKY (W). The concentration-contraction relationship for noradrenaline (NA, 100 pM–1 μM, A), 5-hydroxytryptamine (5-HT, 1 nM–3 μM, B) or carbachol (CCh, 1 nM–3 μM, C). (**A**,**B**) Agonist-induced contraction was normalized to the contraction induced by KCl (72.7 mM). (**C**) CCh was cumulatively applied after the precontraction induced by NA (30 or 300 nM) had reached a steady state, and the contraction was normalized to the NA-induced precontraction. Results were expressed as means ± S.E.M. (n = 5). * *p* < 0.05, ** *p* < 0.01 vs. W (Two-way ANOVA followed by Holm–Sidak post-hoc test).

We next examined the effects of human omentin-1 on agonist-induced contraction and relaxation in isolated thoracic aorta from SHR. First, we examined the contraction induced by noradrenaline (NA, 100 pM–1 μM) or 5-hydroxytryptamine (5-HT, 1 nM–3 μM). In thoracic aortas from W, NA and 5-HT induced contractions in a concentration-dependent manner (Figure 2A,B, n = 5). In thoracic aortas from SS, the NA (Figure 2A, *p* < 0.05 at 100 nM, n = 5)- and 5-HT (Figure 2B, *p* < 0.01 at 1–3 μM, n = 5)-induced contractions were significantly enhanced compared with those in W. Human omentin-1 (18 μg/kg/day, 14 days) did not affect the agonist-induced contractions in isolated thoracic aorta from SHR (Figure 2A,B, n = 5). We then examined the relaxation induced by carbachol (CCh, 1 nM–3 μM). In thoracic aortas from W, CCh induced relaxation on NA (30 or 300 nM)-induced pre-contraction in a concentration-dependent manner (Figure 2C, n = 5). In thoracic aortas from SS, the CCh-induced relaxation was significantly impaired (Figure 2C, *p* < 0.05 at 3 μM, *p* < 0.01 at 30 nM–1 μM, n = 5). Human omentin-1 (18 μg/kg/day, 14 days) did not affect the CCh-induced relaxation in the isolated thoracic aorta from SHR (Figure 2C, n = 5).

### 2.3. Human Omentin-1 Tended to Improve Heart Failure in SHR

We next examined the effects of human omentin-1 on cardiac hypertrophy in SHR. The weight of the whole heart (n = 4, *p* < 0.05) and left ventricle (LV) (n = 4, *p* < 0.01) was significantly higher in SS compared with W (Table 1). Human omentin-1 (18 μg/kg/day, 14 days) had no effect on the heart weight in SHR (Table 1, n = 4). It is worthy of note that human omentin-1 tended to decrease the increased weight of the left atrium (LA) in SHR (Table 1, n = 4).

**Figure 3:**
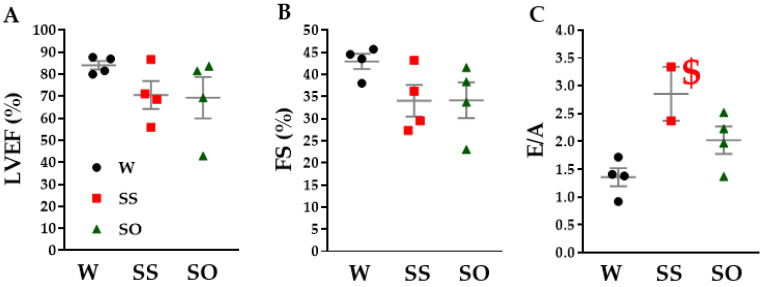
Effects of human omentin-1 on heart failure in SHR. Male SHR (65–68-week-old) were subcutaneously injected with saline (0.14 mL/day; SS) and human omentin-1 (18 μg/kg/day; SO) by using an implanted osmotic pump for 14 days. Male WKY were sham operated (W). (**A**) The left ventricular ejection fraction (LVEF, n = 4, %), (**B**) fractional shortening (FS, n = 4, %), and (**C**) the ratio between velocities of early diastole (E) and late diastole (A) inflow across mitral valves (E/A, n = 2) were measured with echocardiography. The results were expressed as means ± S.E.M. LVEF was calculated via dividing (LVEDV−LVESV) by LVEDV. FS was calculated via dividing (LVDd−LVDs) by LVDd. LVEDV; left ventricular end-diastolic volume, LVESV; left ventricular end-systolic volume, LVDd; left ventricular end-diastolic diameter, LVDs; left ventricular end-systolic diameter. ^$^ E and A wave could not be calculated (n = 2). Statistical evaluations were performed using a One-way ANOVA followed by Bonferroni’s post-hoc test.

We further examined the effects of human omentin-1 on cardiac dysfunction with echocardiography. Left ventricular ejection fraction (LVEF) and fractional shortening (FS) indicating LV systolic function tended to decrease in SS compared with W (Figure 3A,B, n = 4). Human omentin-1 had no effect on the LV systolic parameters in SHR (Figure 3A,B, n = 4). At the same time, the ratio between velocities of early diastole (E) and late diastole (A) inflow across mitral valves (E/A) indicating LV diastolic dysfunction tended to increase in SS compared with W (Figure 3C, n = 2). Human omentin-1 tended to reverse E/A in SHR (Figure 3C, n = 4). We also found a very short deceleration time (n = 4) or the absence of the A wave (n = 2) in SS for which E/A could not be calculated. Human omentin-1 tended to reverse the diastole time in SHR (n = 4).

**Figure 4:**
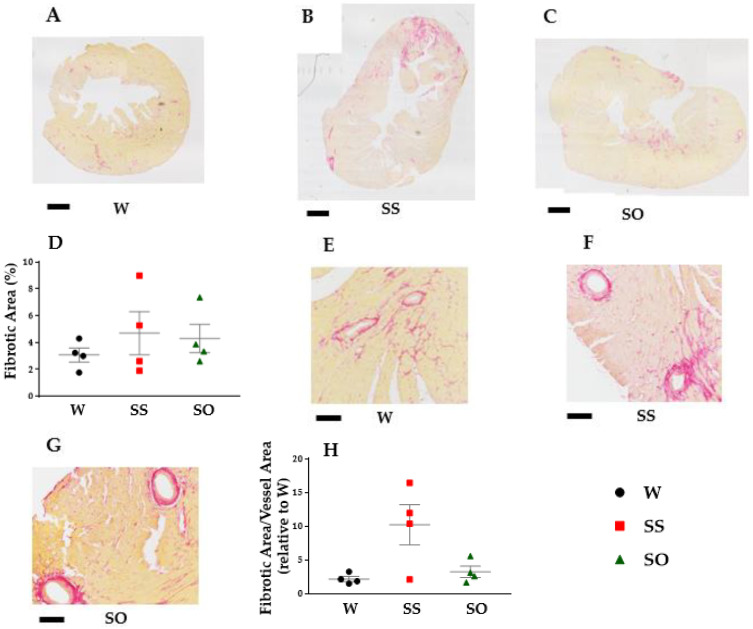
Effects of human omentin-1 on LV fibrosis in SHR. The heart was isolated from male SHR (65–68-weeks-old) subcutaneously injected with saline (0.14 mL/day, n = 4; SS) and human omentin-1 (18 μg/kg/day, n = 4; SO) by using an implanted osmotic pump for 14 days or sham operated male WKY (n = 4; W). (**A**–**C**) The paraffin-embedded LV tissue sections were made and the representative results for picrosirius red staining were shown (A, W; B, SS; C, SO). (**D**) The interstitial fibrotic area (red) in LV tissue was measured with ImageJ software. The ratio (%) of fibrotic area (red) was calculated via dividing the red area by the whole tissue area (red and yellow) in LV. The results were shown as means ± S.E.M. (**E**–**G**) The paraffin-embedded LV tissue sections were made and the representative results for picrosirius red staining specifically focusing on small blood vessels are shown (E, W; F, SS; G, SO). (**H**) The perivascular fibrotic area (red area around the vessels) was corrected by each vessel area. The results were shown as fold increase relative to W (means ± S.E.M). Scale bars indicate 1.0 mm (**A**–**C**) or 0.2 mm (**E**–**G**). Statistical evaluations were performed using a One-way ANOVA followed by Bonferroni’s post-hoc test.

In order to explore mechanisms of protective roles of human omentin-1 on LV diastolic dysfunction, we further examined the effects of human omentin-1 on LV interstitial and perivascular fibrosis in SHR. The interstitial fibrotic area in LV tended to increase in SS compared with that in W (Figure 4A,B, n = 4). Human omentin-1 (18 μg/kg/day, 14 days) tended to decrease it in LV from SHR (Figure 4C,D, n = 4). In addition, the perivascular fibrotic area in LV also tended to increase in SS compared with that in W (Figure 4E,F, n = 4). Human omentin-1 tended to decrease it in LV from SHR (Figure 4G,H, n = 4).

### 2.4. Human Omentin-1 Tended to Improve Renal Dysfunction in SHR

**Figure 5:**
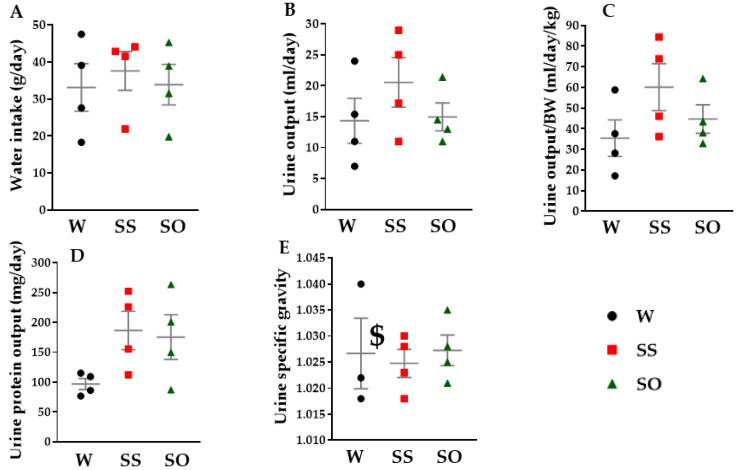
Effects of human omentin-1 on 24 h water intake and urine output in SHR. Male SHR (65–68-week-old) were subcutaneously injected with saline (0.14 mL/day, n = 4; SS) and human omentin-1 (18 μg/kg/day, n = 4; SO) by using an implanted osmotic pump for 14 days. Male WKY were sham operated (n = 4; W). Urine was collected by placing rats in metabolic cages for 24 h. (**A**) Water intake (g/day), (**B**) urine output (ml/day), (**C**) urine output/body weight; BW (mL/day/kg), and (**D**) urine protein output (mg/day) were calculated. Urine protein concentration (mg/mL) was measured with a BCA Protein Assay. (**E**) Urine specific gravity was measured with a urine gravity meter (n = 3). ^$^ One sample was over the measurement limitation. Results were expressed as means ± S.E.M. Statistical evaluations were performed using a One-way ANOVA followed by Bonferroni’s post-hoc test.

We next examined the effects of human omentin-1 on the 24 h water metabolism in SHR. The water intake (Figure 5A, n = 4) and urine output (Figure 5B,C, n = 4) tended to increase in SS compared with W. Human omentin-1 (18 μg/kg/day, 13 days) tended to decrease them in SHR (Figure 5A–C, n = 4). We further examined the effects of human omentin-1 on the biochemical characteristics of urine and blood in SHR. The urine protein output tended to increase in SS compared with W (Figure 5D, n = 4). Human omentin-1 tended to decrease it in SHR (Figure 5D, n = 4). The urine specific gravity tended to decrease in SS compared with W (Figure 5E, n = 4). Human omentin-1 tended to increase it in SHR (Figure 5E, n = 4).

**Figure 6:**
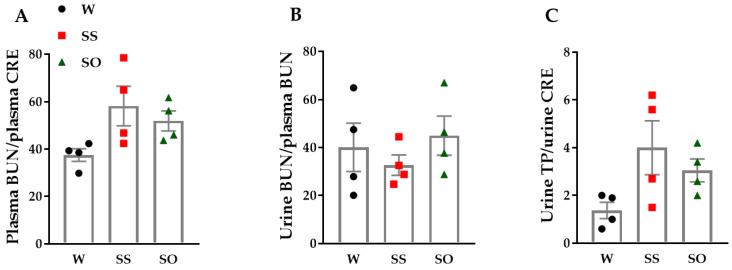
Renal dysfunction in SHR tended to be improved by human omentin-1. Heparin-anticoagulated plasma and urine were collected from male SHR (65–68-week-old) subcutaneously injected with saline (0.14 mL/day, n = 4; SS) and human omentin-1 (18 μg/kg/day, n = 4; SO) by using an implanted osmotic pump for 14 days or sham operated WKY (n = 4; W). Urine was collected by placing rats in a metabolic cage for 24 h. Blood urea nitrogen (BUN, mg/dL) and creatinine (CRE, mg/dl) in plasma and urine were measured with Wet Chemical Analysis. Urine total protein (TP) concentration (mg/mL) was measured with a BCA Protein Assay. (**A**) Plasma BUN/plasma CRE, (**B**) Urine BUN/plasma BUN, and (**C**) Urine TP/urine CRE were calculated. Results were expressed as means ± S.E.M. Statistical evaluations were performed using One-way ANOVA followed by Bonferroni’s post-hoc test.

The plasma blood urea nitrogen (BUN)/plasma creatinine (CRE) tended to increase in SS compared with W (Figure 6A, n = 4). Human omentin-1 (18 μg/kg/day, 14 days) tended to decrease it in SHR (Figure 6A, n = 4). The urine BUN/plasma BUN tended to decrease in SS compared with W (Figure 6B, n = 4). Human omentin-1 tended to increase it in SHR (Figure 6B, n = 4). The urine total protein (TP)/urine CRE tended to be higher in SS compared with W (Figure 6C, n = 4). Human omentin-1 tended to decrease it in SHR (Figure 6C, n = 4).

## 3. Discussion

**Figure 7:**
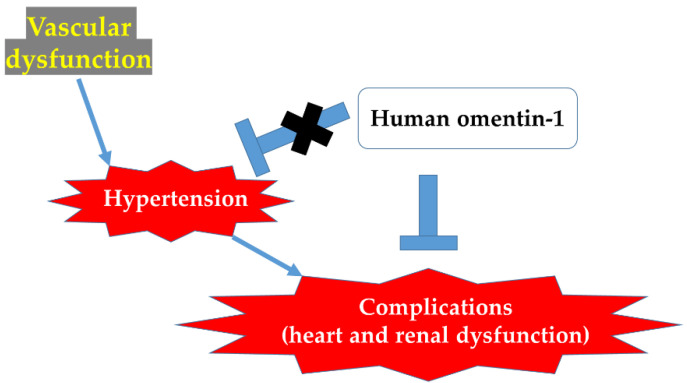
Human omentin-1 tended to improve the hypertension-associated complications (heart and renal dysfunction) in SHR without affecting hypertension.

The major findings of the present study are as follows: (1) Human omentin-1 had no influence on BW, HR, and SBP in SHR; (2) Human omentin-1 had no influence on the enhancement of contractile responses by NA or 5-HT or on the impairment of relaxant response by CCh in isolated thoracic aorta from SHR; (3) Human omentin-1 had no influence on LV hypertrophy and systolic dysfunction, but improved LV diastolic failure and fibrosis in SHR; (4) Human omentin-1 tended to decrease the increased water intake, urine output, urine protein output, and renal failure markers in SHR. In summary, human omentin-1 (18 μg/kg/day, 2 weeks) administration had no influence on severe hypertension (SBP > 200 mm Hg) and enhanced vasocontractile or impaired vasodilator responses in the isolated thoracic aorta from 65–68-week-old SHR. On the other hand, it tended to improve LV diastolic failure and renal failure in SHR. The present study demonstrated for the first time that human omentin-1 tends to reduce the hypertensive complications (heart and renal failure), while it had no influence on severe hypertension in aged SHR (Figure 7).

The dose of human omentin-1 used in the present study (18 μg/kg) is assumed to be approximately 270 ng/mL in blood, based on the estimation that the circulating blood volume is 62 × {BW (kg) + 0.02} (mL) in rats with BW ≥ 120 (g) [15]. Since this concentration was close to 300 ng/mL at which human omentin-1 had previously shown protective effects on cardiovascular cells in vitro [16,17,18], it was determined to be the appropriate dose. It was also reported that the serum concentration of human omentin-1 decreased to 72.19 ± 54.33 ng/mL in stage 1 hypertensive patients (140 ≤ SBP ≤ 159 mm Hg and 90 ≤ diastolic blood pressure; DBP ≤ 99 mm Hg) or to 62.45 ± 47.01 ng/mL in stage 2 hypertensive patients (160 ≤ SBP ≤ 179 mm Hg and 100 ≤ DBP ≤ 109 mm Hg) compared with 147.84 ± 58.55 ng/mL in normotensive humans (SBP ≤ 139 mm Hg and DBP ≤ 90 mm Hg) [8]. Similarly, the blood concentration of omentin-1 in rats was reported to be approximately 90 ng/mL in the serum of Zucker lean rats heterozygous for the mutant leptin receptor gene *fa* (lean Zucker *^fa/+^*), while it decreased to approximately 30 ng/mL in the serum of Zucker Diabetic Fatty rats (ZDF) homozygous for the *fa* mutant gene (obese ZDF *^fa/fa^*) [9]. Since there are no reports on blood omentin-1 concentration in SHR, it is necessary to determine it.

In the present study, human omentin-1 had no influence on severe hypertension in aged SHR (Figure 1). The SHR used in the present study were already at the ages (65–68-week-old) when their survival rate begins to decline [4]. On the other hand, since we could not exclude the possibility that human omentin-1 may suppress mild hypertension, we needed to examine whether the human omentin-1 administration prevents the development of hypertension in younger SHR.

In the present study, we found that human omentin-1 administration tended to decrease the fibrosis of both cardio interstitial and perivascular areas in LV tissues from SHR (Figure 4). Since it was reported that these fibrotic changes in LV were associated with diastolic cardiac failure [19], it is suggested that human omentin-1 may prevent the diastolic dysfunction of LV, perhaps through decreasing LV fibrosis. The activation of transforming growth factor (TGF) pathway was considered as one of the mechanisms for the fibrosis in LV from SHR [20]. Since human omentin-1 was reported to inhibit the TGF pathway [21], human omentin-1 administration may ameliorate the fibrosis in LV from SHR, perhaps through inhibiting the TGF pathway. In addition, we found that human omentin-1 administration tended to prevent the increase in LA weight in SHR (Table 1). Since LA expansion was reported to be a useful predictor for the LV diastolic failure in patients with severe diastolic dysfunction [22], the data that human omentin-1 prevented the increase in LA weight may support its protective role on LV diastolic failure in SHR.

In the present study, human omentin-1 administration tended to inhibit the increase in urine output in SHR (Figure 5). In aged SHR, the urine output was reported to increase, which was related to the bladder dysfunction [4]. Therefore, it is presumable that human omentin-1 administration may inhibit the increased urine output by affecting the bladder function in SHR. In addition, we found that human omentin-1 administration tended to ameliorate renal dysfunction (increasing of BUN/CRE) in SHR (Figure 6). Human omentin-1 administration was reported to protect renal function through anti-inflammatory and anti-oxidative effects in type 2 diabetic nephropathy mice [23]. Therefore, human omentin-1 may ameliorate renal dysfunction through anti-inflammatory and anti-oxidative effects.

It was reported that rat omentin administration increased cyclic GMP levels in the isolated left ventricular tissue of rats [24]. It was also reported that NO administration improved renal blood flow in the patients with cardiac failure [25]. Thus, it seems likely that human omentin-1 might improve cardiac and renal failure in aged SHR via the promotion of the NO pathway.

This study had the following limitations: We were lucky to obtain 9 aged SHR and WKY, and planned to use them at 65–68-weeks of age for this experiment. However, it is very difficult (almost impossible) to get additional SHR and WKY at this age, since the survival rate in SHR was 100% (15/15 rats) at 54-weeks of age; however, it declined to 46.7% (7/15 rats) at 72-weeks of age. In contrast, the survival rate in WKY at 72-weeks of age was 100% (7/7 rats) [4]. On the other hand, we believe that our data using such aged SHR and WKY are very valuable for the same reason.

In conclusion, the present study demonstrates for the first time that human omentin-1 tends to suppress hypertensive complications (heart and renal failure), although it has no influence on severe hypertension in aged SHR. Further studies on human omentin-1 may lead to the developing of therapeutic agents for hypertensive complications. It might also be possible that human omentin-1 is one of the indicators for the risks of cardio-renal organ damage in hypertensive patients.

## 4. Materials and Methods

### 4.1. Materials

The reagent sources were as follows: human omentin-1 (BioVendor R&D, Karasek, Czech Republic), NA, 5-HT, and CCh (Sigma-Aldrich, St. Louis, MO, USA).

### 4.2. Animals

Male WKY and SHR (65–68-week-old) (Hoshino Laboratory Animal Inc., Ibaraki, Japan) were divided into three groups (W: WKY + Sham, SS: SHR + Saline, SO: SHR + Omentin, n = 4/group). A slow-release ALZET osmotic 2 mL pump (Model 2002; Muromachi Kikai Co., Ltd., Tokyo, Japan) was subcutaneously implanted to infuse human omentin-1 or saline for 14 days in SHR. An animal study was approved by the ethical committee of the School of Veterinary Medicine of Kitasato University (approval no. 21-066), and was performed in conformity with an institutional guideline of the Kitasato University. All animals were placed into a room with constant temperature and humidity (22 ± 2 °C, 50–60%, 12 h for lighting). The rats were able to freely take food (CE2, CLEA Japan, Inc., Tokyo, Japan) and tap water.

### 4.3. Measurement of BW, HR, and SBP

The BW, SBP, and HR were measured once a week (before injection and 3–4 and 10–11 days after injection). The tail-cuff system (Softron, Tokyo, Japan) was utilized in conscious conditions for a measurement of HR and SBP as described previously [26,27,28]. More than 10 measurements per 1 rat were performed.

### 4.4. Measurement of Isometric Contraction

WKY (66–68-week-old) and age-matched SHR were deeply anesthetized with urethane (1.5 g/kg, i.p.) and euthanized by exsanguination. The thoracic aorta was isolated from the descending aorta. After the removal of fat and adventitia, ring preparation (2 mm in long) was made as described previously [10,29]. The ring was placed into a 2 mL organ bath filled with normal physiological salt solution (PSS) saturated with 95% O_2_ and 5% CO_2_ mixture at 37 °C and pH 7.4. The smooth muscle contractility was measured isometrically with a force-displacement transducer (Minebea Mitsumi, Nagano, Japan) and recorded as computer data by using the PowerLab system (ADInstruments, New South Wales, Australia). Each ring was hanged on two hooks under a resting tension of 0.5 g in the organ bath and repeatedly exposed to the high K^+^ solution containing 72.7 mM KCl until the responses became stable as described previously [26]. Concentration-response curves to NA (100 pM–1 μM) or 5-HT (1 nM–3 μM) were obtained by the cumulative application. The high K^+^-induced maximal contractions were used for normalization. Concentration-relaxation responses were obtained by the cumulative application of CCh (1 nM–3 μM) after NA (30 or 300 nM)-induced submaximal precontraction became stable.

### 4.5. Measurement of Heart Weight

After the rats were anesthetized with urethane (1.5 g/kg, i.p.) and euthanized by exsanguination, the hearts were harvested. They were washed with PSS, separated into LA, right atrium (RA), LV, and right ventricle (RV), and weighed.

### 4.6. Echocardiographic Examination

Echocardiographic examination was performed on day 11 or 12 after injection with 2–3% isoflurane anesthesia, as described previously [30,31]. In the present study, the rat was positioned in a lateral recumbency. Data were recorded digitally and subsequently analyzed with a commercially available analysis software package supplied with the Echo system (LOGIQ S8, GE Healthcare, Tokyo, Japan). Each two orthogonal left ventricular views were recorded to calculate left ventricular volumes at both end-systole and end-diastole by Simpson’s method. The LVEF was calculated as follows: [left ventricular end-diastolic volume (LVEDV)−left ventricular end-systolic volume (LVESV)/LVEDV] × 100 (%). The short-axis left ventricular view was recorded to measure the left ventricular internal diameter by M-mode. FS was calculated as follows: [left ventricular end-diastolic diameter (LVDd)−left ventricular end-systolic diameter (LVDs)/LVDd] × 100 (%). Doppler inflow across the mitral valve was measured on the apical four-chamber view. The sample volume was positioned at the tip of the mitral valve leaflets. The transmitral flow velocity patterns were traced along the instantaneous highest velocity spectra to determine the peak velocity of the E wave and A wave, and the mean value of three cardiac cycles was used to calculate the E/A.

### 4.7. Picrosirius Red (PSR) Staining

The formalin-fixed LV tissue was embedded in paraffin and thin sections (4 μm) were made for PSR staining as described previously [32]. The images were obtained using a light microscope (BX-S1; OLYMPUS, Tokyo, Japan) equipped with a microscopic digital camera (DP74; OLYMPUS). The interstitial and perivascular fibrotic area in PSR stained LV tissue sections was measured with ImageJ software (Version 1.53a, NIH, Bethesda, MD, USA). The interstitial fibrotic area ratio (%) was calculated via diving red stained PSR-positive area by the whole tissue area in LV. The perivascular fibrotic area in LV tissue sections was corrected by each vessel area.

### 4.8. Measurement of Water Intake, Urine Output, and Urine Specific Gravity

After rats were placed in metabolic cages (SN-78; Shinano, Tokyo, Japan) for 24 h on day 12–13 after the injection, the measurement of water intake and urine output was performed. Urinary specific gravity was measured with a clinical refractometer (ERMA Inc., Tokyo, Japan).

### 4.9. Blood and Urine Chemical Analysis

Urine protein output was measured by a BCA assay kit (FUJIFILM Wako Pure Chemical Co., Ltd., Osaka, Japan). Plasma BUN/plasma CRE, urine BUN/plasma BUN, and urine TP/urine CRE were calculated after measuring the BUN and CRE with a chemistry analyzer (Dimension RXL Max System, SIEMENS Co., Ltd., Tokyo, Japan).

### 4.10. Statistical Analysis

Data are shown as means ± standard error of mean (S.E.M.). Statistical analyses were performed using two-way ANOVA followed by the Holm-Sidak post-hoc test (Figure 1 and Figure 2) or one-way ANOVA followed by the Bonferroni’s post-hoc test (Figure 3, Figure 4, Figure 5, Figure 6 and Table 1). Values of *p* < 0.05 were considered to be statically significant.

## Figures and Tables

**Table 1:** Effects of human omentin-1 on cardiac hypertrophy in spontaneously hypertensive rats (SHR). The heart was isolated from male SHR (65–68-week-old) subcutaneously injected with saline (0.14 mL/day, n = 4; SS) and human omentin-1 (18 μg/kg/day, n = 4; SO) by using an implanted osmotic pump for 14 days or sham operated male Wistar Kyoto rats (WKY) (n = 4; W). The wet weight (g) of the left ventricle (LV), left atrium (LA), right ventricle (RV), and right atrium (RA), as well as tail length (TL, cm) were measured. The LV weight was corrected by TL. Results were expressed as means ± standard error of the mean (S.E.M.). * *p* < 0.05, ** *p* < 0.01 vs. W (One-way ANOVA followed by Bonferroni’s post-hoc test).

	W (n = 4)	SS (n = 4)	SO (n = 4)
Whole heart (g)	1.301 ± 0.009	1.575 ± 0.050 *	1.531 ± 0.082
LV (g)	0.967 ± 0.005	1.286 ± 0.061 **	1.238 ± 0.077 *
RV (g)	0.256 ± 0.007	0.206 ± 0.011 **	0.220 ± 0.006 *
LA (g)	0.034 ± 0.002	0.045 ± 0.005	0.037 ± 0.005
RA (g)	0.044 ± 0.002	0.039 ± 0.001	0.037 ± 0.002
100 × LV/TL	4.955 ± 0.066	7.359 ± 0.349 **	7.333 ± 0.375 **
TL (cm)	19.5 ± 0.4	17.5 ± 0.2 *	16.9 ± 0.6 **

## Data Availability

The detailed data of the current study are available from the corresponding authors upon reasonable request.

## Abbreviations

5-HT	5-hydroxytriptamine
b.p.m.	Beats per minutes
BUN	Blood urea nitrogen
BW	Body weight
CCh	Carbachol
CRE	Creatinine
DBP	Diastolic blood pressure
E/A	the ratio between velocities of early diastole (E) and late diastole (A) inflow across mitral valves
FS	fractional shortening
HR	Heart rate
i.p.	Intraperitoneal
LA	Left atrium
LV	Left ventricle
LVDd	Left ventricular end-diastolic diameter
LVDs	Left ventricular end-systolic diameter
LVEDV	Left ventricular end-diastolic volume
LVEF	Left ventricular ejection fraction
LVESV	Left ventricular end-systolic volume
NA	Noradrenaline
NO	Nitric oxide
PSR	Picrosirius red
PSS	Physiological salt solution
RA	Right atrium
RV	Right ventricle
SBP	Systolic blood pressure
S.E.M.	Standard error of the mean
TGF	Transforming growth factor
TL	Tail length
TP	Total protein
